# The Association Between Delayed Graft Function and Renal Resistive Index in Kidney Transplant Recipients

**DOI:** 10.7759/cureus.17315

**Published:** 2021-08-19

**Authors:** Serkan Bakirdogen, Hasan Anil Kurt, Fatih Kamış, Sibel Bek, Aysegul Erbayraktar

**Affiliations:** 1 Nephrology Department, Canakkale 18 Mart University, Canakkale, TUR; 2 Urology, Canakkale Onsekiz Mart University, School of Medicine, Canakkale, TUR; 3 Internal Medicine, Canakkale Onsekiz Mart University, School of Medicine, Canakkale, TUR; 4 Nephrology, Kocaeli University Hospital, Kocaeli, TUR

**Keywords:** delayed graft function, doppler ultrasound, kidney transplant recipient, post transplantation, renal resistive index

## Abstract

Background and objectives

Delayed graft function (DGF) may increase the risk for kidney graft dysfunction. Renal resistive index (RRI) in Doppler ultrasonography is useful in measuring blood flow changes in kidneys which is indicative of tubulointerstitial damage. Most of the diseases in DGF etiology are related to tubulointerstitium and arteries of the kidneys. In this study, we investigated whether there is a relationship between delayed graft function and renal resistive index in kidney transplant recipients (KTR).

Materials and methods

Patients who underwent kidney transplantation were included in this retrospective study. KTR were divided into two groups as DGF (+) and DGF (−). Comparison of RRI values of DGF (+) and DGF (−) groups according to the measurements at different times.

Results

The findings showed that both RRI measurements (post-transplant in the first week and the end of the first year) of the DGF (+) group were higher than DGF (−) group (p=0.001 and p=0.003, respectively). The interaction of measurements and DGF did not have an effect on RRI (p>0.05).

Conclusion

The value of RRI in the DGF (−) group was lower than DGF (+) group in the first week after kidney transplantation.

## Introduction

Delayed graft function (DGF) is defined as the need for dialysis within the first week after kidney transplantation. The etiology of DGF includes acute tubular necrosis, accelerated/acute rejection, thrombotic microangiopathy, vascular and surgical complications (ureter stenosis, urinary leakage) [1,2]. The effects of DGF on graft survival in the future are controversial. The prognosis may vary according to the underlying etiology. DGF may increase the risk for graft dysfunction and mortality in the late period [1,3-6].

Renal resistive index (RRI) in Doppler ultrasonography is useful in measuring blood flow changes in kidneys and showing damage to the tubulointerstitium [7]. The decrease in renal artery blood flow was associated with prolonged cold ischemia time and the development of DGF [8,9]. Most of the diseases in DGF etiology may affect the tubulointerstitium of the kidneys [1,2]. In this study, we investigated whether there is a relationship between delayed graft function and renal resistive index in kidney transplant recipients (KTR).

## Materials and methods

Our study was designed retrospectively. Patients who underwent renal transplantation in the organ transplant unit of our hospital between October 2015 and March 2020 were included in this study. DGF was defined as the need for dialysis within the first week after kidney transplantation [1,2]. Kidney transplant recipients were divided into two groups as DGF (+) and DGF (−). Retrospective records and renal Doppler ultrasonography data of the patients were collected from the hospital. Renal artery stenosis, renal vein thrombosis, advanced aortic valve regurgitation, heart failure, acute pyelonephritis, and acute rejection were accepted as exclusion criteria for this study. This study started after the approval of the local ethics committee.

RRI value was determined by examining the interlobar artery in the upper, middle, and lower zones of the kidney and by the average of three different measurements. Toshiba Aplio XG Doppler ultrasonography device was used together with a convex transducer (PVT-375BT). RRI was calculated using the formula ‘‘peak systolic velocity − end-diastolic velocity/peak systolic velocity’’ [7]. Doppler ultrasonography was performed on the patients by the specialists in the radiology clinic of our hospital. Two (post-transplant in the first week and end of the first year) RRI measurements of each patient were recorded. 

Blood was drawn from each patient in the early morning following at least eight hours of fasting. All blood samples were studied in our hospital’s biochemistry laboratory. Serum creatinine and urea analyses of the patients were examined using the colorimetric method on the Roche Cobas 6000 device 501 modules. Biochemistry markers of each patient were recorded on the first day of kidney transplantation, the third month, and at the end of the first year.

The data collected from the patients in this study were transferred to the Statistical Package for the Social Sciences (SPSS) 19.0 program (IBM Corp., Armonk, NY). The demographic data of the patients, such as age and gender, were determined by descriptive statistics. Comparison of DGF (+) and DGF (−) groups on the age variable were performed using the Mann-Whitney U test since they did not show normal distribution. The relationship of the patient groups concerning gender was examined by chi-square analysis. Comparison of the groups concerning RRI at post-transplant in the first week was performed by Student’s t-test because it conformed to normal distribution. As the number of patients decreased, the comparison of RRI values of the groups at the end of the first post-transplant year was performed using the Mann-Whitney U test. Bonferroni, a multiple comparison test, was conducted to investigate that there were significant differences between groups. Comparison of RRI, serum urea, and creatinine values of DGF (+) and DGF (−) groups according to the measurements at different times was made using a two-factor analysis of variance (ANOVA) test.

## Results

Eighty-six kidney transplant recipients (29 females, 57 males) were included in this study. The average age of the patients was 52±13.07. Of the kidney transplant recipients, 45 (from 44 cadaveric and one living donor) were DGF (+) and 41 (from 37 cadaveric and four living donors) were DGF (−). When evaluated according to chronic kidney disease, 17 patients had diabetes mellitus, 32 had hypertension, 12 had chronic glomerulopathy, four had autosomal dominant polycystic kidney disease, three had urological causes, and 18 had an unknown etiology. Antithymocyte globulin (1-3 mg/kg/day, 2-14 days) was administered to each renal transplant recipient as induction therapy. Triple immunosuppressive therapy (tacrolimus, prednisolone, and mycophenolate mofetil) was given to each patient after transplantation.

No statistically significant difference was found between the groups regarding age (p=0.792). The findings obtained in this study showed that there was no statistically significant difference in the diagnosis of DGF according to gender (p=0.937). Cold ischemia time was higher in DGF (+) group than in DGF (−) group. The result was statistically significant (p=0.002). The groups were found similar concerning human leukocyte antigen (HLA) mismatch (p=0.081).

**Figure 1:**
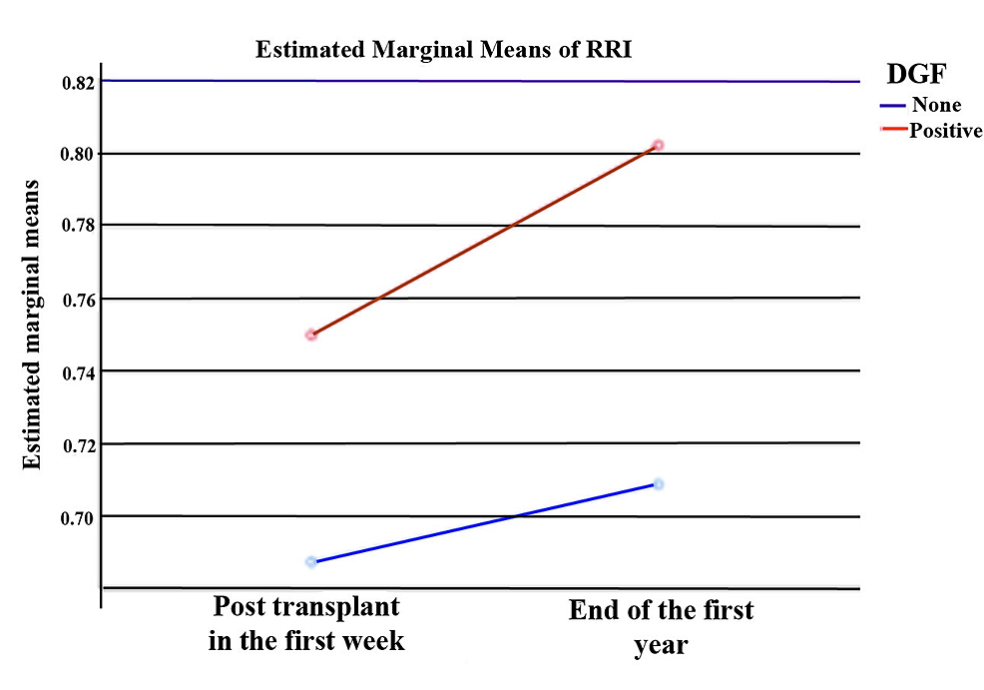
Estimated marginal means of RRI in both groups RRI: renal resistive index.

RRI measurements of DGF (+) and DGF (−) patients in the first week after kidney transplantation and at the end of the first year were compared with each other. The estimated marginal means of RRI in both groups are shown in Figure 1. Both RRI measurements (post-transplant in the first week and end of the first year) of the DGF (+) group were higher than DGF (−) group. The results were statistically significant (p=0.001 and p=0.003, respectively). The comparison of age, cold ischemia time, HLA mismatch, and RRI values of the groups after kidney transplantation are given in Table 1.

**Table 1:** The comparison of age, cold ischemia time, HLA mismatch, and RRI values of the groups after kidney transplantation HLA: human leukocyte antigen, RRI: renal resistive index. *The interaction of measurements.

	Groups	N	Mean±std	Median (min-max)	t	U	p-Value
Age (year)	DGF (−)	41	51.93±12.31	54 (25–74)		892.00	0.792
	DGF (+)	45	52.53±13.86	55 (24–84)			
Cold ischemia time (minute)	DGF (−)	36	805.9±517.7	728 (99–2683)		465.50	0.002
	DGF (+)	44	994.5±406.9	947.5 (240–2670)			
HLA mismatch	DGF (−)	39	4.33±0.93	5 (1-5)		700.00	0.081
	DGF (+)	45	4.07 ±0.96	4 (0-5)			
RRI (1)	DGF (−)	33	0.69±0.07	0.68 (0.43–0.82)	−3.380		*0.001
	DGF (+)	40	0.75±0.08	0.75 (0.55–0.95)			
RRI (2)	DGF (−)	18	0.71±0.09	0.72 (0.55–0.85)		71.00	0.003
	DGF (+)	18	0.80±0.08	0.80 (0.65–0.95)			

RRI values were statistically significantly different according to the measurement (F=5.141, p<0.05). RRI values were statistically significantly different according to DGF groups (F=23.064, p<0.05) but it was seen that the interaction of measurements and DGF did not have a statistically significant effect on RRI (F=0.908, p>0.05). The comparison of the RRI values of DGF groups according to the measurement at different times is shown in Table 2.

**Table 2:** The comparison of the RRI values of the DGF groups according to the measurement at different times (two-factor ANOVA) RRI: renal resistive index, DGF: delayed graft function, ANOVA: analysis of variance. *The interaction of measurements.

Source of variance	F	p	η^2^
Measurement (RRI)	5.141	0.025	0.047
DGF	23.064	0.0001	0.180
Measurement (RRI)* DGF	0.908	0.343	0.009

Serum creatinine levels were not statistically significantly different according to the measurement (F=2.621, p>0.05). Creatinine levels were statistically significantly different according to DGF groups (F=26.95, p<0.05), but it was seen that the interaction of measurements and DGF did not have a statistically significant effect on serum creatinine levels (F=2.109, p>0.05).

**Figure 2:**
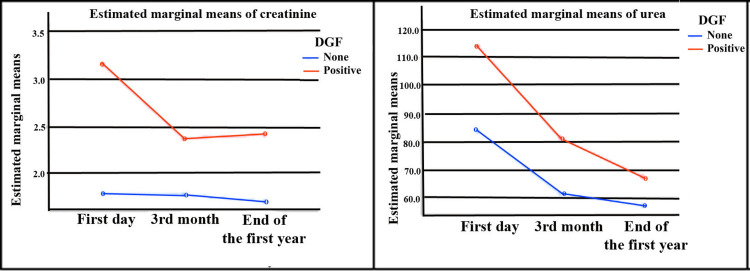
Estimated marginal means of serum urea and creatinine levels in both groups

Serum urea levels were statistically significantly different according to the measurement (F=22.0, p<0.05). Urea levels were statistically significantly different according to DGF groups (F=17.67, p<0.05), but it was seen that the interaction of measurements and DGF did not have a statistically significant effect on serum urea levels (F=1.469, p>0.05). Serum mean urea and creatinine levels and standard deviation values of the groups after kidney transplantation are given in Table 3. The comparison of the mean serum urea and creatinine levels of DGF groups according to the measurement at different times is shown in Table 4. Estimated marginal means of serum urea and creatinine levels in both groups are shown in Figure 2.

**Table 3:** Serum mean urea and creatinine levels and standard deviation values of the groups after kidney transplantation

Measurement (after transplantation)		Creatinine (mg/dL)			Urea (mg/dL)		
		Mean	Standard deviation	N	Mean	Standard deviation	N
The first day	DGF (−)	1.78	0.91	40	83.92	36.82	40
	DGF (+)	3.16	2.02	44	114.04	42.54	44
	Total	2.50	1.73	84	99.69	42.47	84
Third month	DGF (−)	1.77	0.77	40	61.64	29.63	40
	DGF (+)	2.37	1.14	37	81.34	36.83	37
	Total	2.06	1.01	77	71.10	34.52	77
End of the first year	DGF (−)	1.69	0.84	35	57.40	32.51	35
	DGF (+)	2.41	1.49	32	67.53	32.73	32
	Total	204	1.24	67	62.24	32.76	67

**Table 4:** The comparison of the mean serum urea and creatinine levels of the DGF groups according to the measurement at different times (two-factor ANOVA) DGF: delayed graft function, ANOVA: analysis of variance. *The interaction of measurements.

Serum markers	Source of variance	F	p	η^2^
Creatinine	Measurement (creatinine)	2.621	0.075	0.023
	DGF	26.950	0.0001	0.108
	Measurement (creatinine)* DGF	2.109	0.124	0.019
Urea	Measurement (urea)	22.000	0.0001	0.165
	DGF	17.670	0.0001	0.074
	Measurement (urea)* DGF	1.469	0.232	0.013

## Discussion

The risk of developing DGF after kidney transplantation from a donation after brain death (DBD) was reported to be between 24% and 70% [2]. This rate is higher in kidney transplant patients who have undergone donation after cardiac death [2,10]. In our study, people with brain death could become deceased kidney donors. Donations after cardiac death could not be performed because they are not legally possible in our country. DGF was observed in 52.3% of our kidney transplant recipients. There are many diseases in DGF etiology [1,2]. Cold ischemia time is independently associated with the risk of DGF [10]. HLA mismatch is an independent predictor of DGF [11]. However, the functional amino acid polymorphisms in the antigen recognition region of the HLA-A molecule may have an effect on DGF [12]. Cold ischemia time was higher in DGF (+) group than in DGF (−) group in our study. The groups were found similar concerning HLA mismatch (Table 1).

Delayed graft function is defined as the need for dialysis within the first week after kidney transplantation [2]. During this period, patients are usually taken on dialysis due to life-threatening (e.g., hyperkalemia, metabolic acidosis, pulmonary edema, uremic complications) indications [13]. Serum creatinine alone is not ideal for identifying DGF. However, failure of serum creatinine to fall below pre-transplant levels within one week in patients with DGF can also be seen. There is an insufficient number of studies in the literature investigating the effect of serum urea level on DGF development in kidney transplant recipients [14]. In our study, we found higher serum urea and creatinine levels in DGF (+) patients compared to those with DGF (−).

In addition to measuring blood flow changes in the kidneys, RRI also reflects damage to the tubulointerstitium [7]. Many diseases affecting the tubulointerstitium and some vascular complications are included in the etiology of DGF [1,2]. Among kidney transplant recipients with low RRI values have a lower rate of DGF than those with high values [15]. There are several studies in the literature investigating whether RRI has a predictive role on DGF in kidney transplant recipients [16,17]. The elevated RRI is linked to a higher risk of DGF. The predictive efficacy of RRI was higher when the evaluation was done early after transplantation [16]. To predict the development of DGF, the sensitivity of RRI in the intraoperative and postoperative first day is higher [17]. However, this application is not easily applied in the clinic. For this reason, we were able to evaluate the RRI value in kidney transplant recipients within the first week (between one and seven days) after transplantation. In long-term follow-up, the patients with lower RRI values showed better graft survival rates than higher ones [15]. Increased RRI values are seen in patients with DGF, acute rejection, and acute tubular necrosis [18]. RRI values better reflect DGF in the first month after kidney transplantation. However, the RRI value between the first and third months could be due to acute changes in the graft [19]. In our study, we found the RRI value to be higher in DGF (+) group than DGF (−) group in the first week after kidney transplantation. In both groups, the value of RRI in the first week was lower than at the end of the first year. Despite these positive results, the two-factor ANOVA analysis found that RRI was insufficient to predict DGF in kidney transplant recipients.

Our study has some limitations. This study was planned retrospectively. Most of the patients in our center had a deceased kidney donor, and the number of patients transplanted from a living donor was low. Thus, subgroups of patients who underwent kidney transplantation from living and deceased donors could not be compared with each other. RRI values of a small number of patients could be measured due to lack of clinical indication and patient incompatibility. Recipient dialysis status, panel reactive antibodies, the detailed information of the donor's kidney, the kidney donor profile index, and warm ischemia time (donor and recipient) could not be determined.

## Conclusions

The value of RRI in the DGF (−) group was lower than the DGF (+) group in the first week after kidney transplantation. The elevated RRI may be linked to a higher risk of DGF within the first week after transplantation. Prospective studies defining the optimal cut-off value and timing measurement of RRI should be designed in this area.
